# First survey of *Enterocytozoon bieneusi* and dominant genotype Peru6 among ethnic minority groups in southwestern China’s Yunnan Province and assessment of risk factors

**DOI:** 10.1371/journal.pntd.0007356

**Published:** 2019-05-23

**Authors:** Baiyan Gong, Yaming Yang, Xiaohua Liu, Jianping Cao, Meng Xu, Ning Xu, Fengkun Yang, Fangwei Wu, Benfu Li, Aiqin Liu, Yujuan Shen

**Affiliations:** 1 Department of Parasitology, Harbin Medical University, Harbin, China; 2 Department of Helminth, Yunnan Institute of Parasitic Diseases, Puer, China; 3 National Institute of Parasitic Diseases, Chinese Center for Disease Control and Prevention; Chinese Center for Tropical Diseases Research; WHO Collaborating Centre for Tropical Diseases; National Center for International Research on Tropical Diseases, Ministry of Science and Technology; Key Laboratory of Parasite and Vector Biology, MOH; Shanghai, China; Faculty of Science, Ain Shams University (ASU), EGYPT

## Abstract

**Background:**

*Enterocytozoon bieneusi* is the most common microsporidian species causing diarrhea and other intestinal disorders in humans and animals. Like other infectious diseases, microsporidiosis usually disproportionately affects poor populations. In China, some ethnic minority areas remain poor. Currently, no information of *E*. *bieneusi* infection is available in minority populations. The present aims were to understand occurrence and genetic characterizations of *E*. *bieneusi* in ethnic minority groups from a poverty-stricken ethnic township in Yunnan Province, and to assess risk factors for *E*. *bieneusi* infection.

**Methodology/Principal findings:**

289 fecal specimens were collected from Yao people (one specimen each) with and without diarrhea, in Yunnan Province. *E*. *bieneusi* was identified and genotyped by PCR and sequence analysis of the ITS region of the rRNA gene. An average prevalence of 8.30% (24/289) was observed and four genotypes were identified—genotype Peru6 (n = 21) and three novel genotypes (one each). Genotype Peru6 was detected in two family members in each of three families. In a phylogenetic analysis, all of four genotypes fell into group 1 with zoonotic potential. The people owning individual pit toilets had a statistically higher prevalence of *E*. *bieneusi* (16.67%, 12/72) than those using public pit toilets (6.06%, 12/198).

**Conclusions/Significance:**

This is the first report on occurrence and genetic characteristics of *E*. *bieneusi* in ethnic minority groups in China. Genotype Peru6 was found in humans in China for the first time and showed dominance in Yao people. The same genotype was found in some family members and all the genotypes fell into group 1, suggesting the possibility of anthroponotic and zoonotic transmissions. The majority (83.33%, 20/24) of *E*. *bieneusi* positive individuals did not present diarrhea. In any case, it is important to recognize their existence and the importance that asymptomatic individuals to *E*. *bieneusi* may have from an epidemiological point of view, as transmitters of this pathogen. The analysis of risk factors provides scientific evidence for the development of effective strategies for prevention and control of *E*. *bieneusi* infection.

## Author summary

*Enterocytozoon bieneusi* is the most prevalent opportunistic microsporidian species causing diarrhea in humans and animals worldwide. Due to frequent occurrence of chronic or life-threatening diarrhea mainly in HIV/AIDS patients and others patients such as elderly people, transplant recipients and cancer patients, epidemiological studies of *E*. *bieneusi* focus on this susceptible population. Asymptomatic carriers of *E*. *bieneusi* are often neglected, and their role in the transmission of this microorganism is not paid enough attention. The prevalence of *E*. *bieneusi* is also actually related to the geographical locations, the populations and the socioeconomic conditions. The infectious diseases usually disproportionately affect poor populations. In China, although total economy has developed rapidly, there is the regional imbalance in economic development and some minority areas remain poor. Thus, we carried out a molecular epidemiology survey of *E*. *bieneusi* among 289 Yao people with and without diarrhea from a poverty-stricken ethnic township. We identified *E*. *bieneusi* in 8.30% (24/289) of people, with the majority (83.33%, 20/24) of positive individuals having no diarrhea. Meanwhile, zoonotic genotype Peru6 was found to be dominant in infected people. Our study emphasizes the importance and significance in epidemiological surveys of *E*. *bieneusi* in non-diarrheal people and assessment of risk factors for *E*. *bieneusi* infection.

## Introduction

Microsporidia are a group of unicellular, obligate intracellular parasitic eukaryotes which are composed of approximately 1,300 to 1500 formally described species in more than 200 genera [1]. Seventeen species belonging to nine genera have been identified in humans [2]. Among them, *Enterocytozoon bieneusi* shows the highest frequency (more than 90%) in human cases of microsporidiosis [3]. *E*. *bieneusi* causes a disease characterized mainly by diarrhea, which appears to be related to replication of organisms in the villus epithelium of the small intestine, along with reduced villus height and surface area [4]. However, clinical signs and symptoms are variable depending on the health status of infected hosts, and the genetic background and infective doses of this pathogen [3,4]. Usually, asymptomatic infection or self-limiting diarrhea occurs in immunocompetent or healthy individuals while chronic or life-threatening diarrhea in immunocompromised individuals [3].

With accumulation of epidemiological data of *E*. *bieneusi*, the host range of *E*. *bieneusi* has been expanding gradually. In addition to humans, this pathogen has been found in many animal species, revealing the zoonotic nature [5,6]. Meanwhile, infective spores of *E*. *bieneusi* have been identified in some water bodies, such as a drinking source watershed [7], drinking source water [8], surface waters [9,10] as well as raw wastewater in wastewater treatment plants, indicating the possibility of waterborne transmission [11–13]. Relatively few studies have documented the presence of *E*. *bieneusi* in foods (milk, raspberries, beans and lettuce) [14,15]. However, one outbreak of microsporidiosis due to *E*. *bieneusi* has been linked to contaminated food (cucumbers in salad and sandwich) served at conference in a hotel [15]. In fact, human cases of *E*. *bieneus*i infection are mainly through ingesting water and food contaminated by *E*. *bieneusi* spores from human and animal feces. Based on clinical and public health importance, *E*. *bieneusi* has been defined as the second highest priority organisms/biological agents (ranked on category B list) by the National Institutes of Health (NIH) of the USA [13]. Meanwhile, *E*. *bieneusi* is also listed on the Environmental Protection Agency (EPA) microbial contaminant candidate list of concern for waterborne transmission [16].

With application of genotyping tools and development of genetic markers for *E*. *bieneusi*, significant advances have been made in the understanding of the host specificity and evolution of *E*. *bieneusi*, and the transmission routes and clinical picture of human microsporidiosis. Currently, due to a very high degree of genetic polymorphism in the internal transcribed spacer (ITS) region of the ribosomal RNA (rRNA) gene within *E*. *bieneusi*, sequence analysis of the ITS region is regarded as the standard method for species identification and genotyping of *E*. *bieneusi* isolates [17]. To date, more than 360 ITS genotypes have been reported, and at least 70 genotypes have been identified in humans, with 33 of them in both humans and animals [6,18]. By phylogenetic analysis of the ITS sequences of *E*. *bieneusi*, all the genotypes are divided into ten different genetic groups [19]. Group 1 is the largest and most complicated, for this group is composed of more than 94% published genotypes, including almost all the genotypes from humans and the majority of genotypes from animal hosts [20]. Meanwhile, it has been observed in group 1 that some genotypes co-occur in humans and animals, and those only found in animals show a close genetic relationship to human-pathogenic ones, thereby establishing zoonotic potential of these genotypes [19,21]. Groups 2 to 5, 8, 9, 10 and the outlier mostly consist of host-adapted genotypes, and groups 6, 7 contain the genotypes found in urban wastewater in China and AIDS patients in Nigeria, respectively [19].

In China, since the first report of microsporidiosis caused by *E*. *bieneusi* in both humans and animals in 2011 [22], this pathogen has been attracting increased attention. To date, molecular epidemiological studies of *E*. *bieneusi* have been carried out in 26 provinces/ municipalities in various mammal species [6] and some bird species [23,24]. In contrast, *E*. *bieneusi* has only been found in humans in seven provinces/ municipalities, with the prevalence ranging from 0.20% to 22.50% [2,22,25–31]. Meanwhile, only two studies assessed risk factors for human *E*. *bieneusi* infection [25,26]. China is a multi-ethnic country. There are great differences among different ethnics and regions when it comes to culture, economy, customs and natural environments. Some ethnic minority areas are comparatively backward economically and culturally, which possibly have influence on the prevalence of *E*. *bieneusi* in humans. Currently, no reports about occurrence and genetic characterizations of *E*. *bieneusi* in minority populations are available in China. Thus, a cross-sectional epidemiological survey of *E*. *bieneusi* was carried out to understand prevalences and genetic characterizations of *E*. *bieneusi* in Yao people from a poverty-stricken ethnic township in Yunnan Province by PCR and sequence analysis of the ITS region of the rRNA gene. Meanwhile, possible risk factors for *E*. *bieneusi* infection were assessed in the investigated areas.

## Materials and methods

### Ethics statement

The present study protocol was reviewed and approved by the Ethics Committee of the National Institute of Parasitic Diseases, Chinese Center for Disease Control and Prevention, China (ethics application no. 201401). Before beginning work, we orally explained our study objectives and procedures to all participants, and obtained their permission to have their fecal specimens involved in our study. Meanwhile, written informed consents were signed by each participant. Each head of the selected households was responsible for giving them to us.

### Study area

Yunnan Province is located in the far southwest of China, bordering with the countries Vietnam, Laos and Myanmar. Our study was carried out in Mengla County of Xishuangbanna Dai Autonomous Prefecture, Yunnan Province (geographical coordinates: 21.27°N latitude, 101.33°E longitude). Mengla County has a tropical savanna climate with strong monsoonal influences. The annual average temperature is 22.1°C, with the lowest (averaging 18.1°C) in December and the highest (averaging 29.9°C) in June. Yao Ethnic Township is one of poverty-stricken ethnic townships in Mengla County. Local sanitation infrastructure is inadequate: only one public latrine in each village, no garbage centralized dumping sites and domestic sewage drainage facility. Domestic pigs and chickens are the most common animals in villages, with chickens roaming freely. Although piped water had been supplied by the end of 2010, most villagers do not break a poor hygiene habit of eating unwashed vegetables and fruit.

### Study population and collection of fecal specimens

In April, 2018, 289 fresh fecal specimens (approximately 5–10 g) were collected from Yao people (one specimen each) inhabiting in three minority villages of Yao Ethnic Township in Mengla County, with 93, 110 and 86 from Guangming, Nangongshan, and Suoshanjiao villages, respectively. The number of people involved in the present study accounted for approximately 35% of total population of each village. All the participants were immunocompetent adults (range, 21–72 years) with 43 suffering from diarrhea at the time of sampling. Meanwhile, a structured questionnaire including demographic information and possible risk factors for *E*. *bieneusi* infection was administered to each participant (Table 1). All the fecal specimens were transported to the laboratory in a cooler with ice packs and stored in refrigerators at -20°C prior to being used in molecular biological characterizations.

**Table 1:** Assessment of possible risk factors for *E*. *bieneusi* infection in Yao people.

Variable		Examined no. ^b^	Positive no. (%)	OR (95% CI ^c^)	χ^2^/*P*-value
Gender	Male	153	10 (6.54)	0.61 (0.26, 1.42)	1.34/0.25
	Female	136	14 (10.29)		
Diarrhea	Yes	43	4 (9.30)	0.86 (0.28, 2.66)	0/1.00
	No	246	20 (8.13)		
Drinking boiled water	Yes	258	23 (8.91)	0.93 (0.12, 7.52)	0/1.00
	No	12	1 (8.33)		
Washing hands before meals	Yes	224	21 (9.38)	0.67 (0.19, 2.36)	0.11/0.74
	No	46	3 (6.52)		
Washing hands after using toilets	Yes	242	23 (9.50)	0.35 (0.05, 2.72)	0.48/0.49
	No	28	1 (3.57)		
Eating unwashed vegetables and fruits	Yes	236	23 (9.75)	0.28 (0.04, 2.15)	0.96/0.33
	No	34	1 (2.94)		
Swimming	Yes ^a^	40	1 (2.50)	4.33 (0.57, 33.03)	1.53/0.22
	No	230	23 (10.00)		
Pit toilet	Public	198	12 (6.06)	0.32 (0.14, 0.76)	**7.33/0.007** ^**d**^
	Individual	72	12(16.67)		
Feeding patterns of animals	Free-ranging	50	3 (6.00)	0.51 (0.08, 3.34)	0.04/0.85
	Both free-ranging and captive	202	19 (9.41)	0.83 (0.18, 3.89)	0/1.00
	Captive	18	2 (11.11)	Ref	

^a^ Swimming in ponds in the investigated areas.

^b^ Only 270 participants provided complete information other than gender and clinical signs (diarrhea).

^c^ CI, confidence interval.

^d^ Bold type for values indicates statistical significance.

### DNA extraction

DNA extraction was performed on all 289 stored fecal specimens. Genomic DNA of *E*. *bieneusi* was extracted directly from 180 to 200 mg of each fecal specimen using the recommended procedures and the provided reagents by the manufacture of QIAamp DNA Stool Mini Kit (QIAGEN, Hilden, Germany). To obtain high yield of DNA, the lysis temperature was increased to 95°C according to the manufacturer’s suggestion. DNA was eluted in 200 μL of AE and stored at −20°C before it was used for PCR analysis.

### PCR amplification and sequencing

*E*. *bieneusi* was identified and genotyped by nested PCR amplification of an approximately 410 bp nucleotide fragment of the rRNA gene including 243 bp of the ITS region [32]. TaKaRa Taq DNA Polymerase (TaKaRa Bio Inc., Tokyo, Japan) was used for all PCR reactions. A negative control (no DNA water control) and a positive control (DNA of a sheep-derived genotype COS-IV) were used in all PCR tests. PCR was performed at least twice for all DNA preparations. All secondary PCR products were subjected to electrophoresis in a 1.5% agarose gel and visualized by staining the gel with GelStrain (TransGen Biotech., Beijing, China). The detailed protocols were available on the given link: dx.doi.org/10.17504/protocols.io.yvjfw4n.

### Nucleotide sequencing and analyzing

All secondary PCR products of expected size were directly sequenced with a set of primers used for the secondary PCR after being purified on an ABI Prism 3730 XL DNA Analyzer by Sinogeno-max Biotechnology Co., Ltd. (Beijing, China), using the Big Dye Terminator v3.1 Cycle Sequencing Kit (Applied Biosystems, USA). Sequence accuracy was confirmed by sequencing two separate PCR products of the same DNA preparation in both directions. Nucleotide sequences obtained in the present study were subjected to BLAST searches (http://www.ncbi.nlm.nih.gov/blast/), and then aligned and analyzed with each other and reference sequences downloaded from GenBank by using the program Clustal X 1.83 (http://www.clustal.org/). All the genotypes were identified only based on 243 bp of the ITS region of the rRNA gene of *E*. *bieneusi* according to the established nomenclature system [17]. The *E*. *bieneusi* genotypes having the sequences identical to published ones were given the first published name. In contrast, the genotypes, which produced different sequences from published ones and were confirmed by sequencing another two PCR products, were considered to be novel genotypes. All of the novel genotypes were given genotype names by adding specimen codes behind YN (the abbreviation of Yunnan Province).

### Phylogenetic and statistical analyses

To better present the genetic diversity of all the genotypes of *E*. *bieneusi* obtained in this study and to assess the phylogenetic relationship of the novel ones here to the known ones, a neighboring-joining tree of the ITS sequences was constructed using Mega 5 software (http://www.megasoftware.net/), based on evolutionary distances calculated by a Kimura 2-parameter model. The reliability of the tree was assessed by the bootstrap analysis with 1,000 replicates. A nucleotide sequence of *E*. *bieneusi* from a dog (GenBank: DQ885585) was used as outgroup in phylogenetic analysis.

Pearson chi-square (χ^2^) test based on the Statistical Package for the Social Sciences (SPSS) 19.0 were used to determine the relationships between prevalence of *E*. *bieneusi* and the variables listed in Table 1. Differences were considered statistically significant at a *P* value of < 0.05.

## Results

### Prevalence

All 289 fecal specimens were screened for the presence of *E*. *bieneusi* by PCR amplification of the ITS region of the rRNA gene. Twenty-four PCR-positive specimens were successfully sequenced and confirmed the presence of *E*. *bieneusi* by sequence analysis. An average prevalence of 8.30% (24/289) was observed in Yao people in the investigated areas. *E*. *bieneusi* was found in all the three villages investigated. The highest prevalence occurred in the Yao people from Guangming Village (11.83%, 11/93), followed by Nangongshan Village (8.18%, 9/110) and Suoshanjiao Village (4.65%, 4/86). There was no statistical difference in the prevalence of *E*. *bieneusi* among them (*P* > 0.05) (Table 2).

**Table 2:** Prevalence and genotype distribution of *E*. *bieneusi* in Yao people by village.

Location	Examined no.	Positive no. (%)	Genotype/s (n)	
			known	novel
Guangming Village	93	11 (11.83)	Peru 6 (11)	
Nangongshan Village	110	9 (8.18)	Peru 6 (8)	YN104 (1)
Suoshanjiao Village	86	4 (4.65)	Peru 6 (2)	YN241 (1), YN249 (1)
Total	289	24 ^a^ (8.30)	Peru 6 (21)	YN104 (1), YN241 (1), YN249 (1)

^a^ Four with diarrhea and 20 without diarrhea in 24 positive samples.

### Assessment of possible risk factors

In the present study, 289 dispensed questionnaires were all retrieved. However, only 270 questionnaires provided complete information, and they were finally used to assess possible risk factors for *E*. *bieneusi* infection listed in Table 1. Although prevalences of *E*. *bieneusi* differed within each of the group categories of risk factors, the people owning individual pit toilets were observed to have a statistically higher prevalence of *E*. *bieneusi* (16.67%) than those using public pit toilets 6.06% (*P* < 0.01) (Table 1).

### Genotyping and phylogenetic analysis

**Fig 1:**
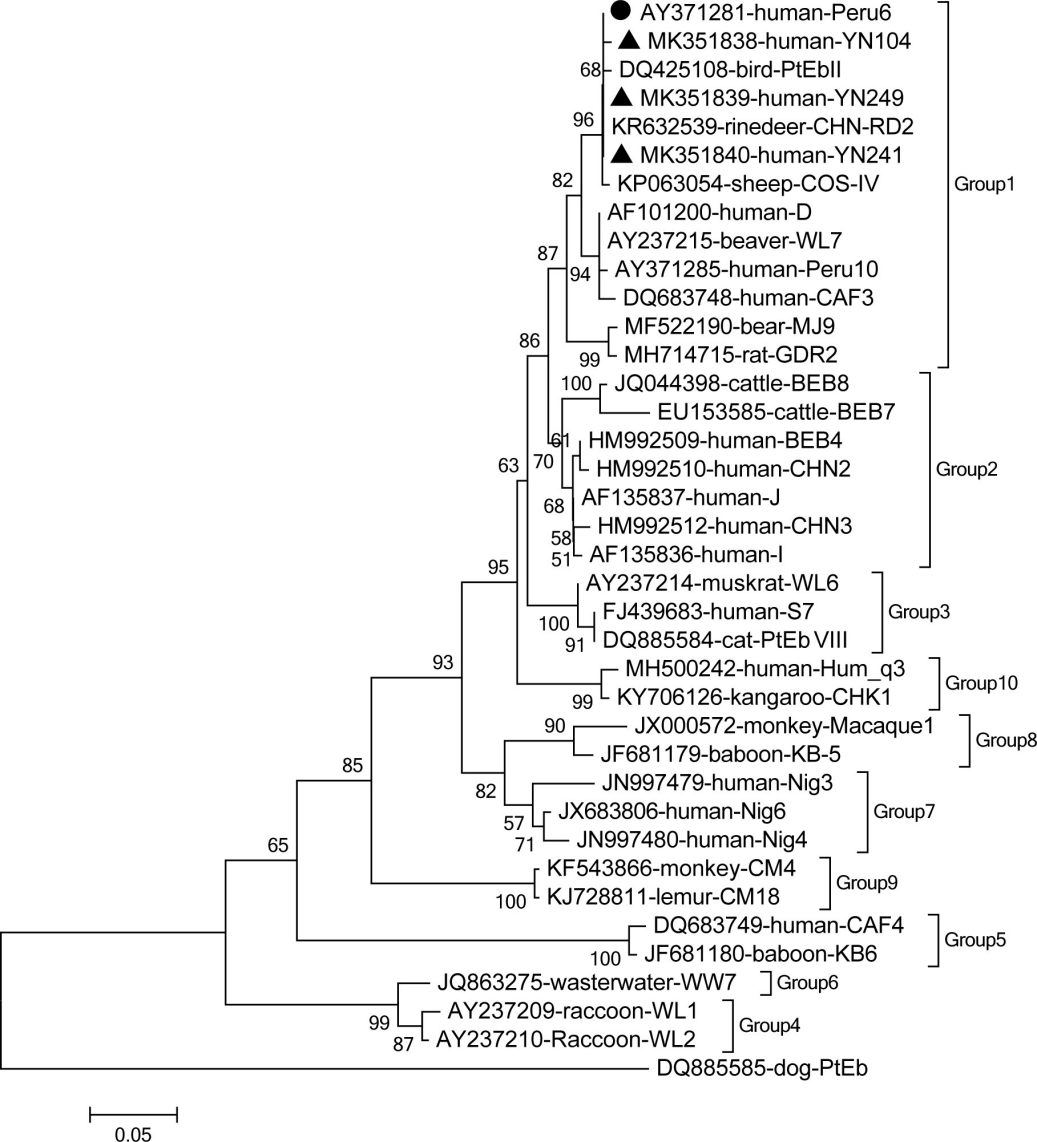
Phylogenetic relationships of the genotypes of *E*. *bieneusi*. The relationships of the genotypes of *E*. *bieneusi* identified in this study and known genotypes published in GenBank were inferred by a neighbor-joining analysis of ITS sequences based on genetic distances calculated by the Kimura 2-parameter model. The numbers on the branches are percent bootstrapping values from 1000 replicates. Each sequence is identified by its accession number, host origin, and genotype designation. The group terminology for the clusters is based on the work of Zhang et al. [19]. The circle and the triangles filled in black indicate known genotype Peru6 and novel genotypes identified in this study, respectively.

Based on sequence analysis of the ITS region of the rRNA gene of 24 *E*. *bieneusi* isolates, four genotypes were identified with four polymorphic sites being observed among them. Twenty-one sequences were identical to each other and had 100% homology with that of genotype Peru6 from a human (GenBank: AY371281). The remaining three sequences were different from each other and were not reported previously. They were considered to be novel genotypes named as YN104 (GenBank: MK351838), YN241 (GenBank: MK351840) and YN249 (GenBank: MK351839). The three genotypes all had the largest similarity with genotype Peru6: one base substitution at position 26 (A to G) for genotype YN104 and at position 103 (A to G) for genotype YN241 and two base deletions at positions 51 and 52 for genotype YN249. In a phylogenetic analysis, all four genotypes obtained here were clustered into zoonotic group 1 (Fig 1).

Genotype Peru6 showed an absolute dominance in Yao people and a wide distribution in the investigated areas. This genotype could be detected in 87.5% of 24 people positive for *E*. *bieneusi* and in all the three villages involved in this study. Meanwhile, two family members were found to be infected with genotype Peru6 in each of three families.

### Relationship between diarrhea and *E*. *bieneusi* genotypes

Genotype Peru6 was identified in three and 18 diarrheal and non-diarrheal cases, respectively. Genotypes YN241 and YN249 were detected in two non-diarrheal cases (one each) while genotype YN104 in one diarrheal case. By statistical calculation, no relationship was found between diarrhea and *E*. *bieneusi* genotypes (*P* > 0.05).

## Discussion

In the present study, by PCR and sequence analysis of the ITS region of the rRNA gene, 8.30% (24/289) of Yao people were found to be positive for *E*. *bieneusi*, with different prevalences being found in the three villages investigated (11.83%, 8.18% and 4.65%). Since the first report of human cases of *E*. *bieneusi* infection in China in 2011, epidemiological studies of *E*. *bieneusi* have been carried out in six different populations, including HIV/AIDS patients (5.71–11.58%), HIV-negative patients (4.25%), cancer patients (1.31%), diarrheal adults (13.25%), diarrheal children (0.20–22.50%), and children without gastrointestinal diseases (4.2–7.5%) [2,22,25–31] (Table 3). In fact, prevalences are complicated and difficult to compare due to differences in the diagnostics methods employed, the specimens analyzed, the geographical locations, the populations investigated, and even individual characteristics (gender, age, socioeconomic conditions, immune status, and clinical features) [3].

**Table 3:** Prevalences and genotypes of *E*. *bieneusi* in humans in China.

Location	Population	Positive no./Examined no. (%)	Genotype (no.)	Zoonotic genotype (no.)	% zoonotic genotypes	Ref
Chongqing	Diarrheal children	11/93 (11.83)	PigEBITS5 (3), CC2 (2), CQ-H1 (1), CQ-H2 (2), CQ-H3 (2), CQ-H4 (1)	PigEBITS5 (3), CC2 (2)	45.45	[29]
Guangxi	HIV-positive patients	33/285 (11.58)	EbpC (4), D (11), PigEBITS7 (7), type IV (8), GX25 (1), GX456 (1), GX458 (1)	EbpC (4), D (11), PigEBITS7 (7), type IV (8)	90.90	[25]
Henan	HIV-positive patients	39/683 (5.71)	EbpC (18), D (7), type IV (6), PigEBITS7 (1), Peru8 (1), EbpD (1), Henan-I-V (1 each)	EbpC (18), D (7), type IV (6), PigEBITS7 (1), Peru8 (1), EbpD (1), Henan-I (1), Henan-III-V (1 each)	97.44	[26]
	HIV-negative patients	29/683 (4.25)	EbpC (21), D (5), Peru11 (1), type IV (1), Unknown (1)	EbpC (21), D (5), Peru11 (1), type IV (1)	96.55	[26]
Heilongjiang	Children without gastrointestinal diseases ^a^	19/255 (7.45) ^b^	EbpC (11), CS-4 (2), Henan-IV (3), NEC1–5 (1 each)	EbpC (11), CS-4 (2), Henan-IV (3)	76.19	[31]
	Cancer patients	5/381 (1.31)	D (4), HLJ-CP1 (1)	D (4)	80.00	[2]
Jilin	Diarrheal children	9/40 (22.50) ^b^	**BEB4 (5)** ^d^, **CHN2 (2), CHN3 (4)**, CHN4 (3), **I (3)**, **J (3)**	**BEB4 (5)** ^d^, **CHN3 (4)**, CHN4 (3), **I (3)**, **J (3)**	90.00	[22]
Shanghai	Children without gastrointestinal diseases	24/573 (4.18)	EbpC (1), D (1), Peru11 (6), EbpA (2), SH1 (1), SH2 (3), SH3 (1), SH4 (1), **BEB6 (1)** ^d^, SH6 (1), Henan-I (1) ^d^, SH8–11 (1 each), type IV (1) ^d^	EbpC (1), D (1), Peru11 (6), EbpA (2), SH2 (3), **BEB6 (1)** ^d^, **Henan-I (1)** ^d^, SH8 (1), type IV (1) ^d^	70.83	[30]
	Diarrheal children	23/169 (13.61) ^c^				[27]
	Diarrheal adults	11/83 (13.25) ^c^				[27]
Hubei	Diarrheal children	1/500 (0.20)	D (1)	D (1)	100	[28]
Total		204/3745 (5.45)	EbpC (55), D (29), PigEBITS7 (8), Peru11 (7), type IV (16), BEB4 (5), CHN2 (2), CHN3 (4), CHN4 (3), I (3), J (3), CS-4 (2), EbpA (2), NEC1–5 (1 each), EbpD (1), Peru8 (1), Henan-I (2), Henan-II (1), Henan-III (1), Henan-IV (4), Henan-V (1), SH1 (1), SH2 (3), SH3 (1), SH4 (1), BEB6 (1), SH6 (1), SH8–11 (1 each), GX25 (1), GX456 (1), GX458 (1), HLJ-CP1 (1), PigEBITS5 (3), CC2 (2), CQ-H1 (1), CQ-H2 (2), CQ-H3 (2), CQ-H4 (1), Unknown (1)	EbpC (55), D (29), PigEBITS7 (8), Peru11 (7), type IV (16), BEB4 (5), CHN3 (4), CHN4 (3), I (3), J (3), CS-4 (2), EbpA (2), EbpD (1), Peru8 (1), Henan-I (2), Henan-III (1), Henan-IV (4), Henan-V (1), SH2 (3), BEB6 (1), SH8 (1), PigEBITS5 (3), CC2 (2)	85.79	

The bolded genotypes belong to group 2.

^a^ Only four children had diarrhea in the study conducted by Yang et al [31].

^b^ Mixed infections were found in the two studies conducted by Yang et al. [31] and Zhang et al. [22].

^c^ *E*. *bieneusi* was only identified based on sequence analysis of the SSU rRNA gene in a study conducted by Liu et al. [27].

^d^ Four genotypes in Table 3 have been changed to the first published names instead of genotype names described in original papers (SH5 to BEB6, CHN1 to BEB4, SH12 to type IV, SH7 to Henan-I).

*E*. *bieneusi* is one of common causative agents of opportunistic infection. Immunodeficiency/immunocompromised individuals, especially that associated with HIV/AIDS, younger age, elderly people, transplant recipients and cancer patients are considered risk factors for intestinal microsporidiosis due to *E*. *bieneusi* [2,3]. Since the first recognition of *E*. *bieneusi* in HIV/AIDS patients in 1985 [33], this pathogen has been reported in HIV-seropositive adults with diarrhea, with the prevalences high up to 78% in developed countries (the USA) [34], and 51% in developing countries (Zimbawe) [35]. There have been some studies reporting high prevalences of *E*. *bieneusi* in children, such as 76.9% in HIV-positive children in Uganda [36], and 22.5% in diarrheal children in China [22]. Personal hygiene habits and environmental sanitation conditions are possibly related to *E*. *bieneusi* infection. In two studies conducted in China, drinking boiled water and hand washing before meals were considered to be protective factors against *E*. *bieneusi* infection in HIV/AIDS patients [25,26]. In a study of molecular epidemiologic characterization of *E*. *bieneusi* conducted in Nigeria, HIV-infected persons using a stream/river as a source of water were observed to have more opportunity to be infected with *E*. *bieneusi* than using treated (tap) water, borehole and well/rain [37]. In fact, the infectious diseases usually disproportionately affect poor populations. In China, although total economy has developed rapidly, there is the regional imbalance in economic development, and some minority areas remain poor. In our analysis of multiple possible risk factors for *E*. *bieneusi* infection in Yao people from a poverty-stricken ethnic township, the people using individual pit toilets were observed to have a statistically higher prevalence of *E*. *bieneusi* (16.67%) than those using public pit toilets (6.06%). This might be related to the fact that local public pit toilets are cleaned and disinfected regularly and feces are disposed of harmlessly according to the method regulated by Sanitary Standard for the Non-hazardous Treatment of Night Soil (GB 7959–87). In contrast, management of individual pit toilets is relatively poor in these aspects and the toilets are close to people’s living environment, increasing the opportunity of cross transmission of *E*. *bieneusi* infection between family members. Based on our analysis result of risk factors, the present primary task is to make prevention and control strategies targeting possible risk factors to reduce *E*. *bieneusi* infection in local inhabitants. Meanwhile, health education should be carried out to make people aware of the severity of this disease and the importance of public health.

By sequence analysis of the ITS region of the rRNA gene of 24 *E*. *bieneusi* isolates, four genotypes (Peru6, and three novel YN104, YN241 and YN249) were identified in the present study, with genotype Peru6 showing an absolute dominance (87.50%, 21/24). In a phylogenetic analysis, all of them fell into zoonotic group 1. It has been reported that almost all human-pathogenic genotypes belonged to zoonotic group 1 in addition to a few in other five groups, such as I, J, BEB4, CHN2, CHN3 in group 2, S7 in group 3, CAF4 in group 5, Nig3, Nig4, and Nig6 in group 6 and Hum_q3 in group 10 [19,22,37–40] (Fig 1). In China, to date, a total of 45 genotypes of *E*. *bieneusi* have been identified in humans, composed of 39 in group 1 and six in group 2; meanwhile, 85.79% of genotypes have been found in animals [2,22,25–31] (Table 3).

Genotype Peru6 obtained in the present study was first reported in humans in Peru in 2003 [41]. Currently, at least 39 human cases of microsporidiosis caused by genotype Peru6 have been reported, distributing in Peru (n > 4), Portugal (n = 14) and China (n = 21) (this study) [41–45]. Besides humans, genotype Peru6 has been detected in eight and nine species of mammals and birds, respectively in Portugal, China, Brazil, Poland and the United States [18,24,46–57] (Table 4). Meanwhile, this genotype has ever been found in wastewater in China [11,58]. These findings above indicated that genotype Peru6 had a broad host range and the possibility of zoonotic and waterborne transmissions. In the investigated areas, there are a lot of domestic animals raised, and chickens can be seen here and there. Combined with the observation of genotype Peru6 in chickens previously [55], chickens were suspected to be source of genotype Peru6 infection in Yao people in the investigated areas. In the present study, some family members were observed to be infected with genotype Peru6, indicating the possibility of person-to-person transmission. To date, evidence of a person-to-person transmission of *E*. *bieneusi* was suggested only in two previous studies conducted in a Thai orphanage based on the fact that only *E*. *bieneusi* genotype A was detected in all positive specimens, and this genotype was found only in humans [59,60]. Therefore, the true burden of human microsporidiosis caused by *E*. *bieneusi* attributable to humans and animals as well as the transmission dynamic of this disease needs to be assessed in the future based genotyping data of *E*. *bieneusi* from local humans and animals, especially in local common domestic animals (pigs) and poultries (chickens).

**Table 4:** Host range and geographical distribution of genotype Peru6 of *E*. *bieneusi*.

Host	Country	Positive no.	Ref
Human	Portugal	14	[45]
	Peru	>4^a^	[41–44]
	China	21	This study
Mammals			
brown rat	China	2	[47]
giant panda	China	3	[48–50]
rabbit	China	1	[18]
reindeer	China	6	[51]
goat	China	3	[46]
sheep	China	5	[46]
cattle	America	1	[52]
dog	Portugal	1	[53]
Birds			
pigeon	Portugal	18	[54]
lovebird	Portugal	1	[54]
chicken	Brazil	1	[55]
rock pigeon	Brazil	2	[56]
rook	Poland	1	[57]
duck	China	2	[24]
goose	China	3	[24]
pigeon	China	22	[24]
red-crowned crane	China	2	[24]
Wastewater	China	7	[11,58]
Total		>120	

^a^ unspecific case number of genotype Peru6 in one study conducted in Peru by Cama et al. [42].

Diarrhea is the most common clinical sign associated with *E*. *bieneusi* infection, especially in immunocompromised individuals. In fact, significant relationships have been observed between *E*. *bieneusi* infection and diarrhea occurrence in HIV-positive individuals in some previous studies [61–63]. Although there are a number of studies reporting the presence of *E*. *bieneusi* in immunocompetent people, infected individuals usually do not show clinical signs [3]. In the present study, similar prevalences were found among diarrheal and non-diarrheal individuals (9.30% versus 8.13%), and there was no relationship between diarrhea and *E*. *bieneusi* genotypes. The occurrence and the severity of diarrhea caused by *E*. *bieneusi* are actually related to multiple factors, including the immune status of the infected hosts, the infective doses of this pathogen and the virulence of genotypes [3]. Here, all the fecal specimens were from immunocompetent individuals, and only common qualitative PCR assay was performed in the detection of *E*. *bieneusi*. At this time, it is difficult to analyze and assess the pathogenicity of microsporidia infection caused by *E*. *bieneus*i at species and genotype levels.

### Conclusions

The present study provided the first report on occurrence and genetic characteristics of *E*. *bieneusi* in ethnic minority groups in China. Genotype Peru6 was found in humans in China for the first time and showed dominance in Yao people in the investigated areas. The findings that the same genotype was found in family members from the same family, and all the genotypes fell into group 1, reveal the possibility of anthroponotic and zoonotic transmissions. High percentage of non-diarrheal people (83.33%, 20/24) emphasizes the importance and significance in epidemiological surveys of *E*. *bieneusi* in this population. In fact, due to no apparent clinical signs and symptoms, these people are largely neglected. Thus, they have more time to continually shed infective spores through feces into the environment, increasing the opportunity of spreading this infection to humans or animals. In addition, local unscientific practices (such as untreated feces as fertilizer on crop and tillage land) can also result in further environmental contamination, and the spores in feces can enter streams and rivers after a heavy rainfall and cause water contamination in new nearby geographical areas. Currently, it is imperative to strengthen management of human feces and surveillance of *E*. *bieneusi* in water bodies including drinking water in the investigated and nearby areas to intervene with and prevent the occurrence of outbreaks of microsporidiosis caused by *E*. *bieneusi*.

In China, despite recent advances in understanding the prevalence and genotypes of *E*. *bieneusi* in humans, studies of possible risk factors for *E*. *bieneusi* infection are insufficient, and the transmission patterns and dynamics of *E*. *bieneusi* are still unclear. Due to China’s vast territory and complicated geographical conditions as well as the regional imbalance in economic development, systematic molecular epidemiological surveys of *E*. *bieneusi* need to be carried out in the future in different populations from broader geographical areas as well as in various animal hosts (e.g. pigs) and environmental specimens (e.g. drinking water).

### Nucleotide sequence accession numbers

Nucleotide sequences of three novel ITS genotypes of *E*. *bieneusi* obtained in the present study were deposited in the GenBank database under the following accession numbers: MK351838 to MK351840.

## Supporting information

S1 Checklist (DOC)
